# Comparing CPAP masks during initial titration for Obstructive Sleep Apnea Syndrome: one-year experience

**DOI:** 10.1016/j.bjorl.2021.10.007

**Published:** 2021-11-25

**Authors:** Adriane Iurck Zonato, Cíntia Felicio Adriano Rosa, Luciana Oliveira, Lia Bittencourt

**Affiliations:** a Hospital IPO — Instituto Paranaense de Otorrinolaringologista, Curitiba, PR, Brazil; b Universidade Federal de São Paulo (UNIFESP), São Paulo, SP, Brazil

**Keywords:** CPAP, Titration, Mask, Nasal pillow, Oronasal

## Abstract

**Objective:**

Continuous Positive Airway Pressure (CPAP) is the gold standard treatment for OSA. A wide variety of masks are used during CPAP therapy. The aim of the study is to compare polysomnography outcomes during titration for different types of masks (oronasal, nasal and pillow) and assess the impact on PAP titration.

**Methods:**

We retrospectively analyzed all CPAP titration polysomnography for one year. Anthropometric data and baseline apnea severity were documented.

**Results:**

A total of 497 patients were evaluated. Nasal masks were used in 82.3% (n = 409), pillow in 14.1% (n = 70) and oronasal in 3.6% (n = 18). There was no difference according to body mass index and baseline apnea-hypopnea index among the groups. The oronasal group was older and had higher titrated CPAP pressure, higher residual apnea-hypopnea index, lower sleep efficiency, more superficial N1 sleep and longer wake time after sleep onset. All other polysomnography outcomes were similar among the groups. The mean CPAP level was 11.6 ± 2.1 cm H_2_O for the oronasal mask, 10.1 ± 2.1 cm H_2_O for the nasal mask and 9.8 ± 2.2 cm H_2_O for the pillow. The residual apnea-hypopnea index was 10.4 ± 7.9 for the oronasal mask, 5.49 ± 5.34 events/h for the nasal mask and 4.98 ± 5.48 events/h for the pillow. The baseline apnea-hypopnea index was correlated with of a higher CPAP pressure for all the groups (*p* < 0.001 for the nasal group, *p* = 0.001 for the pillow group and *p* = 0.049 for the oronasal group). Body mass index and residual AHI were correlated with of a higher CPAP pressure for the nasal and pillow groups only (*p* < 0.001).

**Conclusion:**

The interface can have a significant impact on the effectiveness of PAP titration. Patients with oronasal masks have higher CPAP pressure, higher residual apnea-hypopnea index, lower sleep efficiency and higher wake time after sleep onset. Oronasal masks should not be recommended as the first choice for apnea patients.

**Level of evidence:**

: Level 3.

## Highlights

• Little attention has been paid to the effect of different mask styles on the effectiveness of PAP titration.
• Recent advancements in mask designs provide better mask fit and more choices for patients, which may provide more positive CPAP experiences.
• There is a concept that the oronasal mask increases upper airway resistance compared to the nasal masks.
• The use of nasal or nasal pillow masks should be encouraged by sleep laboratories as the first choice on the CPAP titration night.

## Introduction

Obstructive Sleep Apnea (OSA) is characterized by repeated episodes of obstructive respiratory events during sleep. The airflow limitation results in oxygen desaturation and sleep fragmentation. Untreated OSA is associated with significant morbidity and mortality, with increased risk of hypertension, cardiac arrhythmias, depression, motor vehicle accidents and premature death.^1^, ^2^

Patients with OSA are treated with Continuous Positive Airway Pressure (CPAP), which is delivered during sleep, and it is considered the gold standard treatment from moderate to severe cases. The level of pressure can be titrated through polysomnography when delivering CPAP in a sleep laboratory, where the patient is seen by a technician who is an expert in polysomnography and who will choose the type of mask to be used with CPAP. There are two main types of masks: those placed over the nose and those placed over the nose and the mouth at the same time. There are two nasal mask styles: nasal itself and nasal pillow. Nasal masks cover exclusively the nose while not compressing the nose wings by being placed immediately above the upper lip and near the eye angle. Nasal pillow masks are two intranasal cushions that have emerged as an alternative to the nasal mask for being smaller and having less contact with the face. The oronasal mask covers the nose and the mouth, and it enables the patient to breathe through the nose and the mouth.

The choice of CPAP delivery interface usually occurs based on the clinician’s personal experience or patient preference.^3^ Recent advancements in mask designs provide better mask fit and more choices for patients, which may provide more positive CPAP experiences. The interface can have a significant impact on acceptance and adherence to CPAP therapy. Sleep laboratories often try different mask styles until one is found that makes the patient reasonably comfortable during a PAP titration study. There is a growing concern that oronasal masks may compromise CPAP effectiveness to treat OSA, but oronasal masks are frequently used because of excessive mouth leak while using nasal masks. Oronasal masks are associated with higher CPAP level, higher residual AHI and poorer adherence compared to nasal masks.^3^ Borel observed that oronasal masks may negatively impact CPAP adherence and recommends their use should be restricted to cases of nasal mask failure.^4^ There is a concept that the oronasal mask increases upper airway resistance compared to the nasal masks. The oronasal mask could push the chin and the tongue backward, consequently inducing an upper airway obstruction.^5^, ^6^

A few studies have looked at compliance with different types of PAP masks.^7^ Little attention has been paid to the effect of different mask styles on the effectiveness of PAP titration. Ebben et al. compared the efﬁcacy of three different masks, nasal pillows, nasal masks and full-face (oronasal) masks, during a single night of titration with CPAP. CPAP applied through nasal pillows and nasal masks was equally effective in treating mild, moderate and severe sleep apnea.^8^

This study compared oronasal, nasal and nasal pillow masks during CPAP titration night. We studied the polysomnography outcomes during CPAP titration and assessed whether patient’s characteristics (anthropometric data) differed among the mask preference groups. The aim of the study is to compare polysomnography outcomes during titration for different types of masks (oronasal, nasal and nasal pillow) and assess the impact on the effectiveness of PAP titration.

## Methods

### Study design and subjects

We retrospectively studied all the patients with OSA who have consecutively submitted to CPAP titration for one year, from May 2017 to April 2018, during our clinical practice at the sleep center of our institution.

The following data were recorded for all the patients: age, sex, Body Mass Index (BMI) and baseline Apnea-Hypopnea Index (AHI). The inclusion criteria were age > 18 years old, diagnosed with OSA who underwent polysomnography for CPAP titration. The exclusion criteria were patients under 18 years old; patients who required bi-level positive airway pressure (Bilevel); submitted to automatic titration; submitted to split-night study, patients who switched masks during the titration; patients without baseline AHI; patients with central sleep apnea; patients with severe pulmonary disease (PCD or hypoventilation); patients with severe heart disease (CHF) and patients with dementia; with no information by technician regarding the mask style; and by technical mistake.

### Polysomnography

All the patients underwent full-night CPAP titration with standard polysomnography according to the American Academy of Sleep Medicine standards. Multi-channel recordings of the electroencephalogram (frontal, central and occipital), Electrooculogram (EOG), Electromyogram (EMG), oronasal flow (by mask), respiratory effort (by abdominal and thoracic strain gauges), oxygen saturation (pulse oximetry), snoring and body position were recorded on a computerized workstation (Alice 5, Respironics).

Effective pressure was attained when evidence of apneas, hypopneas and snoring were ameliorated according to AASM guidelines^9^ (Kushida). Respiratory events were classified according to published guidelines from the American Academy of Sleep Medicine Scoring Manual 2016, version 2.3 (hypopnea was scored when the peak signal excursions drop by ≥ 30% of pre-event baseline using PAP device flow for ≥ 10 seconds in association with either ≥3% arterial oxygen desaturation or an arousal)^10^ (Berry).

A sleep technician manually titrated pressure. All patients used the Philips-Respironics REMstar device. Heated humidification was provided for all the patients in the study. All the titration studies were reviewed by a single board-certified sleep specialist and the final prescribed CPAP level for each study was determined. Effective pressure was attained when evidence of apneas, hypopneas and snoring were ameliorated.

### Mask choice

Education and mask fitting were performed preceding the titration study by the attending technician. For all the types of interfaces, adjustments were made until the patient felt comfortable and a proper fit was obtained. Nasal masks were fitted using the sizing gauge, while nasal pillows were fitted using side straps. The size of the nasal pillow was reassessed after placement and changed if needed based on adequate seal. Patients were placed on CPAP for a few minutes while awake and could switch to another type of mask. Additional adjustments were made as needed during the laboratory titration to minimize leak and maximize comfort. The recommendation was to start off with a nasal or nasal pillow mask, while switching to an oronasal mask only if the patient was unable to breathe nasally or had substantial mouth leak, after the use of chin straps during the titration to prevent mouth leak. Oronasal masks were used only if the patient had great difficulty breathing through the nose and/or was unable to keep their mouth closed.

The trial was approved by the Ethical Review Board of our institution (CEP:2.676.587). The study was not supported by any sponsor.

### Statistical analysis

The results of the quantitative variables were described by means, standard deviations, medians, minimum and maximum values. For the categorical variables, frequencies and percentages were presented. To compare the three groups defined by the mask type against the baseline AHI variable, the one-way analysis of variance (ANOVA) was used. Comparisons were made using the covariance analysis model (ANCOVA) for other quantitative variables, while including baseline AHI as a covariate. To assess the correlation between quantitative variables and CPAP pressure, Pearson linear correlation coefficients were estimated. Data on quantitative variables that did not meet the normal condition were submitted to a logarithmic transformation; *p* < 0.05 values indicated statistical significance. Data were analyzed with the use of Stata/SE v.14.1. StataCorpLP, USA computer program.

## Results

Data were evaluated from 698 consecutive patients for whom CPAP therapy was recommended. According to the exclusion criteria, 201 patients were not included in the analysis: patients under 18 years old (n = 05); with no information regarding the mask style (n = 01); patients who required bi-level positive airway pressure (BiPAP) (n = 26); submitted to split-night study (n = 76); patients with central sleep apnea (n = 08); patients with pulmonary diseases (n = 05); patients without baseline AHI (n = 69); and technical mistake (n = 11). Finally, data from 497 patients were analyzed. Nasal masks were used in 82.3% (n = 409), nasal pillow masks in 14.1% (n = 70) and oronasal masks in 3.6% (n = 18).

The characteristics of the study groups are shown in Table 1, Table 2. The oronasal group was older when compared to the nasal and pillow groups. There were more males in the nasal and oronasal groups (*p* = 0.008). There was no difference between the groups regarding body mass index.

**Table 1:** Patients’ characteristics according to mask used during titration.

	Mask	n	Mean	Median	Minimum	Maximum	SD	p*
Age	Nasal	409	53.4	53.0	20.0	87.0	12.9	
	Pillow	70	56.1	58.0	31.0	84.0	11.0	
	Oronasal	18	62.9	65.5	35.0	85.0	15.2	0.003
BMI	Nasal	409	30.7	30.0	20.1	49.7	4.7	
	Pillow	70	30.9	30.4	24.0	44.2	4.7	
	Oronasal	18	30.6	28.7	25.0	40.0	4.5	0.936

Age: Nasal × Pillow: *p* = 0.099; Nasal × Oronasal: *p* = 0.002; Pillow × Oronasal: 0.043.

**Table 2:** Gender distribution according to mask type.

Gender	Mask type		
	Nasal	Pillow	Oronasal
Female	127	35	6
	31.1%	50.0%	33.3%
Male	282	35	12
	68.9%	50.0%	66.7%
Total	409	70	18
*p*-Value: 0.008			

The titration polysomnography data are shown on Table 3. There was no difference regarding baseline AHI among the groups. The oronasal group had higher the oronasal group was older and had higher titrated CPAP pressure, higher residual AHI, lower sleep efficiency, longer Wake time after Sleep Onset (WASO) and a higher percentage of N1 sleep stage. All the other polysomnography outcomes were similar among the mask groups. The mean CPAP level was 11.6 ± 2.1 cm H_2_O for the oronasal mask; 10.1 ± 2.1 cm H_2_O for the nasal mask and 9.8 ± 2.2 cm H_2_O for the nasal pillow. The residual apnea-hypopnea index (residual AHI) was 10.4 ± 7.9 events/h for oronasal mask; 5.49 ± 5.34 events/h for nasal mask and 4.98 ± 5.48 events/h for nasal pillow.

**Table 3:** Comparison among masks in CPAP titration polysomnography.

Variable	Mask	n	Mean	Median	Minimum	Maximum	SD	*p*
Baseline AHI	Nasal	409	47.9	41.0	5.2	138.0	27.0	
	Pillow	70	43.8	40.5	7.3	116.6	24.1	
	Oronasal	18	54.3	52.0	9.2	116.0	27.0	0.269
CPAP pressure	Nasal	408	10.1	10.0	6.0	15.0	2.1	
	Pillow	70	9.8	9.8	6.0	15.0	2.2	
	Oronasal	18	11.6	12.0	7.0	14.0	2.1	0.016
TST/total sleep time	Nasal	409	345.6	355.0	115.5	473.5	61.5	
	Pillow	70	340.0	352.8	145.5	447.0	63.2	
	Oronasal	18	316.4	322.8	231.0	423.0	60.7	0.114
Sleep efficiency	Nasal	409	79.8	81.7	31.2	98.1	12.6	
	Pillow	70	79.0	82.6	41.0	96.0	12.8	
	Oronasal	18	70.6	70.8	50.1	89.1	12.2	0.008
WASO	Nasal	409	68.7	58.0	4.5	275.0	48.8	
	Pillow	70	70.3	56.5	2.5	224.5	50.7	
	Oronasal	18	100.6	104.3	33.0	205.0	52.1	0.023
Residual AHI	Nasal	409	5.49	3.90	0.00	35.10	5.34	
	Pillow	70	4.98	3.50	0.00	27.80	5.48	
	Oronasal	18	10.4	7.9	1.3	33.5	7.9	0.005
REM AHI	Nasal	397	4.49	2.80	0.00	38.40	5.23	
	Pillow	68	3.93	2.25	0.00	17.50	4.43	
	Oronasal	18	5.69	3.45	0.00	37.50	8.55	0.997
Central AI	Nasal	409	1.67	0.60	0.00	29.10	2.85	
	Pillow	70	1.24	0.65	0.00	8.60	1.66	
	Oronasal	18	3.81	1.05	0.00	26.70	6.91	0.098
Arousal index	Nasal	409	8.83	7.20	0.60	42.70	6.31	
	Pillow	70	8.61	7.45	0.50	28.70	5.61	
	Oronasal	18	11.07	8.35	3.00	25.10	6.52	0.243
N1	Nasal	409	2.87	2.40	0.10	23.00	2.04	
	Pillow	70	2.41	2.20	0.20	8.30	1.48	
	Oronasal	18	3.7	3.1	0.8	9.4	2.4	0.023
N2	Nasal	409	54.5	54.5	19.7	92.2	12.3	
	Pillow	70	53.8	53.1	26.9	90.2	12.2	
	Oronasal	18	55.3	56.6	29.1	75.7	12.5	0.717
N3	Nasal	409	23.6	23.4	0.0	53.2	9.6	
	Pillow	70	24.8	24.5	0.8	44.8	8.8	
	Oronasal	18	22.9	20.2	7.1	41.3	10.1	0.560
REM	Nasal	409	18.9	19.2	0.0	43.2	8.2	
	Pillow	70	19.0	19.2	0.0	41.8	8.8	
	Oronasal	18	18.0	16.0	3.0	34.4	7.7	0.760

CPAP pressure: Nasal × Pillow: *p* = 0.332; Nasal × Oronasal: *p* = 0.001; Pillow × Oronasal: 0.001; EF (sleep efficiency): Nasal × Pillow: *p* = 0.625; Nasal × Oronasal: *p* = 0.002; Pillow × Oronasal: 0.011; WASO: Nasal × Pillow: *p* = 0.804; Nasal × Oronasal: *p* = 0.007; Pillow × Oronasal: *p* = 0.020; AHI CPAP: Nasal × Pillow: *p* = 0.126; Nasal × Oronasal: *p* = 0.001; Pillow × Oronasal: *p* < 0.001; N1: Nasal × Pillow: *p* = 0.069; Nasal × Oronasal: *p* = 0.069; Pillow × Oronasal: *p* = 0.011.

WASO, wake time after sleep onset; Central AI, central apnea index; N1, N1 sleep stage; N2, N2 sleep stage; N3, N3 sleep stage.

The baseline apnea-hypopnea index was positively correlated with CPAP pressure for all the groups (Table 4). Body mass index was positively correlated with CPAP pressure only for the nasal and pillow groups (*p* < 0.001).

**Table 4:** Correlations with CPAP pressure.

Variables	Nasal			Pillow			Oronasal		
	n	r^a^	*p*	n	r^a^	p	n	r^a^	*p*
CPAP pressure × age	408	-0.07	0.152	70	-0.02	0.844	18	0.14	0.572
CPAP pressure × BMI	408	0.41	<0.001	70	0.57	<0.001	18	0.40	0.099
CPAP pressure × baseline AHI	408	0.44	<0.001	70	0.40	0.001	18	0.47	0.049

a Pearson correlation coefficient.

## Discussion

Oronasal mask appeared with the intention of attending patients with oronasal breathing and represents 25% to 45% of the masks used according some studies.^1^, ^4^ One single type of mask would be more manageable and convenient for some sleep laboratories, which made the oronasal mask to be routinely chosen during CPAP titration night. However, oronasal masks can be the most difficult ones to be adjusted to the patient’s face (the contact area with the skin is greater compared to the nasal mask) and there is great possibility of air leak (due to mouth movements during sleep).

Even though oronasal masks are frequently used by sleep laboratories during CPAP titration night, in our results only 3.6% of the patients studied used oronasal masks, which shows that the oronasal mask was an exception choice in our data. This could be related to the fact that technicians in our sleep laboratory are trained and guided to perform selective and careful acclimation, while encouraging patients to use the nasal/nasal pillow mask. Even patients with mouth breathing or nasal obstruction history were encouraged by technicians to try and test the nasal/nasal pillow mask; only patients who did not feel comfortable while using the nasal/nasal pillow mask were then tested with the oronasal.

Our result shows that once the patient is encouraged to use the nasal or nasal pillow mask, most of them continue to use that style during titration night. Our data also show that most patients who used oronasal masks were male and older (average of 62 years old). According to other studies, when given the option to choose from, most patients preferred the nasal mask to the oronasal one.^11^, ^12^ There is evidence that the prolonged use of nasal CPAP reduces mouth opening and oral breathing.^13^, ^14^, ^15^ Therefore, even patients with OSA who claim to be mouth breathers could be initiated with nasal masks.

According to our data, the oronasal mask performed worse in the titration polysomnography when compared to the nasal and nasal pillow masks in the following aspects: titrated CPAP pressure was higher, sleep efficiency was lower, patients were awake for longer, patients had more superficial non-REM N1 sleep, and residual AHI was higher. According to studies and a meta-analysis that compared the impact of nasal and oronasal CPAP, the treatment of OSA with oronasal masks is associated with a signiﬁcantly higher CPAP level (+1.5 cm H_2_O, on average), a signiﬁcantly higher residual AHI (+2.8 events/h) and lower adherence (−48 min/night).^2^, ^7^ Similar to our study, the mean CPAP level was 11.6 ± 2.1 cm H_2_O for the oronasal mask; 10.1 ± 2.1 cm H_2_O for the nasal mask and 9.8 ± 2.2 cm H_2_O for the nasal pillow. Residual AHI was 10.4 ± 7.9 events/h for the oronasal mask; 5.49 ± 5.34 events/h for the nasal mask and 4.98 ± 5.48 events/h for the nasal pillow.

When it comes to the CPAP pressure level, the factors correlated with a higher CPAP pressure were BMI and baseline AHI for the nasal mask and nasal pillow groups, as were also demonstrated by Deshpande et al.^1^ In those two groups, the most obese and most severe patients needed higher levels of CPAP pressure. As for the patients who used the oronasal mask, there was an association with the baseline polysomnography AHI only.

In a recently published study with the analysis of 358 patients who underwent polysomnography for CPAP titration, the differences that the type of mask used can have in the final exam result stand out.^1^ The authors showed that despite the small influence of age, AHI and obesity in the CPAP pressure, the main determinant regardless of pressure was the type of mask used. This can affect the patient’s comfort and the subsequent adherence to the CPAP treatment.

Another study that reported 4 cases of patients who underwent CPAP titration also showed differences among those patients, while considering the type of mask used.^16^ The authors retrospectively reviewed the case histories of the 4 patients who used oronasal masks followed by nasal masks. The 4 cases showed a high residual AHI (43 ± 14.2 events/h) and high CPAP pressure (14.9 ± 6.6 cm H_2_O) with an oronasal mask.

The change to nasal mask significantly reduced the residual AHI (3.1 ± 1.5 events/h). It was shown that in two out of the four cases, the control of respiratory events was possible with a much lower level of CPAP pressure compared to oronasal masks. These authors suggest that for patients that use CPAP with oronasal masks and have residual apnea and/or need a surprisingly high CPAP pressure level, the choice of the mask should be reevaluated and the change to a nasal mask should be considered.

## Conclusion

In our experience, oronasal masks represent a low percentage of interface used during CPAP titration. Patients with oronasal masks have higher titrated CPAP pressure, higher residual AHI, lower sleep efficiency, more superficial N1 sleep and higher WASO during titration. The use of nasal or nasal pillow masks should be encouraged by sleep laboratories as the first choice on the CPAP titration night.

## Authors’ contributions

Adriane Iurck Zonato and Cíntia Felicio Adriano Rosa participated in data collection and manuscript writing; Luciana Oliveira and Lia Bittencourt participated in critical manuscript revision. All authors had substantial contributions to drafting the article or revising it critically for important intellectual content and approved the final version of this article.

Ethics approval

All procedures performed in studies involving human participants were following the ethical standards of the institutional and/or national research committee and with the 1964 Helsinki declaration and its later amendments or comparable ethical standards. The study was a retrospective trial and was approved by the Ethical Review Board of our institution.

## Conflicts of interest

All authors have no actual or potential conflicts of interest to disclose, including financial interests and relationships.
